# Network Analysis of Transcription Factors for Nuclear Reprogramming into Induced Pluripotent Stem Cell Using Bioinformatics

**Published:** 2013-11-20

**Authors:** Chiranjib Chakraborty, Sanjiban S.Roy, Minna J.Hsu, Govindasamy Agoramoorthy

**Affiliations:** 1 Department of Bio-Informatics, School of Computer and Information Sciences, Galgotias University, Greater Noida, India; 2 School of Computing Science and Engineering, VIT University, Vellore, India; 3 Department of Biological Sciences, National Sun Yat-Sen University, Kaohsiung, Taiwan; 4 College of Pharmacy and Health Care, Tajen University, Yanpu, Taiwan

**Keywords:** Gene Network, Nuclear Reprogramming, Transcription Factors, Computational Biology

## Abstract

**Objective:**

Research related to induce pluripotent stem (iPS) cell generation has increased rapidly in recent years. Six transcription factors, namely OCT4, SOX2, C-MYC, KLF4, NANOG, and LIN28 have been widely used for iPS cell generation. As there is a lack of data on intra- and inter-networking among these six different transcription factors, the objective of this study is to analyze the intra- and inter-networks between them using bioinformatics.

**Materials and Methods:**

In this computational biology study, we used AminoNet, MATLAB to examine networking between the six different transcription factors. The directed network was constructed using MATLAB programming and the distance between nodes was estimated using a phylogram. The protein-protein interactions between the nuclear reprogramming factors was performed using the software STRING.

**Results:**

The relationship between C-MYC and NANOG was depicted using a phylogenetic tree and the sequence analysis showed OCT4, C-MYC, NANOG, and SOX2 together share a common evolutionary origin.

**Conclusion:**

This study has shown an innovative rapid method for the analysis of intra and inter-networking among nuclear reprogramming factors. Data presented may aid researchers to understand the complex regulatory networks involving iPS cell generation.

## Introduction

Specific somatic cells can transform into induced pluripotent stem cells (iPS) by introducing transcription factors for nuclear reprogramming (1-4). After selecting various combinations from 24 transcription factors, Takahashi and Yamanaka (1) concluded that the over-expression of four factors (OCT4, SOX2, C-MYC, and KLF4) efficiently reprogram fibroblasts such that they can form colonies of cells morphologically akin to embryonic stem (ES) cells. These colonies also proliferate in a similar way to ES cells (5). Another study showed that an overlapping set of four factors (OCT4, SOX2, NANOG, and LIN28) are sufficient to reprogram human somatic cells to pluripotent stem cells (6). Six common nuclear reprogramming factors (OCT4, SOX2, KLF4, C-MYC, NANOG, and LIN28) are extensively used for generating iPS cells. However, it is possible to reprogram somatic cells with three transcription factors OCT4, SOX2 and KLF4, excluding c-MYC15 as it is naturally oncogenic (7,8). Although, the efficiency is reported to be low (7).

Among the octamer transcription factors, OCT4, also known as POU domain class (5 transcription, factor 1), is an important family member (9). SOX2, known as SRY (sexdetermining region Y-box 2) is a transcription factor crucial for maintaining self-renewal of undifferentiated ES cells (10). Also, another Krüppel-like factor (KLF4) has been linked to cellular functions involving development, proliferation, differentiation, and apoptosis (11). The transcription factor C-MYC is a DNA binding protein, which is associated with processes like cell-cycle regulation, proliferation, growth, differentiation and metabolism (12). NANOG is associated with cell regulatory process like ES-cell self-renewal and pluripotency (13). LIN28 and LIN-28 homolog A protein facilitate expression of the pivotal pluripotency factor OCT4 at the post-transcriptional level (14). With the advancement of bioinformatics network development, the analysis of proteins has become a significant area of research for the discovery of new drugs. Protein–protein interactions can provide a clear representation of the complicated relationships between the proteins (15). Such protein–protein interactions can be represented through network development. In turn, the network analysis of proteins provides scientists with a quantitative framework to investigate large complex networks using bioinformatics (16). Both intra- and inter-network analysis can be performed for proteins to understand how amino acids are related to proteins as well as to understand relations across proteins (17). Such analysis can determine protein structures (18), hydrophobic, hydrophilic regions (19), and functional residues (20). On the other hand, the inter-network analysis can show proteomics information including the protein cascades (21).

The interactive protein networking between the protein cascades can validate *in vitro* as well as *in vivo* targets for future drug development (22). However, data are lacking on the network analyses of six common nuclear reprogramming factors; OCT4, SOX2, KLF4, C-MYC, NANOG and LIN28. Therefore, this study has addressed this gap for the first time by performing a rapid silico network analysis of these nuclear reprogramming factors to depict the connection among the amino acids and to visualize the protein– protein relationships hypothetically. The intra network analysis was done using 2D and 3D models to determine the connection between amino acids. A phylogenetic tree was created to explore the inter network analysis. Network development and analyses between the nuclear reprogramming factors were performed by using bioinformatics tools, algorithm analysis and mathematical modeling.

## Materials and Methods

This bioinformatics study was performed at VIT University (Vellore, India) in collaboration with the Galgotias University (Greater Noida, India).

### Data collection

The first step toward the development and analyses of intra and inter networks among the transcription factors is the listing of human proteins and related genes. Therefore data on 6 nuclear reprogramming transcription factors; OCT4, SOX2, NANOG, LIN28, KLF4 and C-MYC and their genes were pooled from the National Center for Biotechnology Information (NCBI) database (www.ncbi.nih.nlm.gov). The functional protein sequences in FASTA format for these genes were also collected from the same database (23).

### Development of intra-networking structures, phylogenetic tree and monophyletic grouping

The AminoNet (www.bioinformatics.org/aminonet/ AminoNet.html) is a Java-based software tool widely used to construct contact networks among amino acids (24). It can be used to generate the intra-network of a protein and also calculate the values of various topological parameters. This study used “.pdb” files to generate the intranetworking of transcription factors. Based on sequence alignment results, a phylogenetic tree was constructed using the software ClustalW (www. ebi.ac.uk/clustalw) (25) that depicted the distances between the protein sequences. Monophyletic grouping was performed to assess the common ancestor (26,27).

### Protein-protein network

The directed network was modeled using MATLAB (7.3 version) programming and the distance between nodes was estimated using a phylogram, a type of phylogenetic tree. An algorithm was also constructed for the generation of this network. Protein-protein interactions between the nuclear reprogramming factors were explored using the software STRING (http:// string-db.org/). STRING is a widely used database and web resource dedicated to explore the protein-protein interactions, including physical and functional interactions (28).

### Development of sub-network and analysis of strongly connected components

A sub-network of the nuclear reprogramming factors was created from the protein-protein network using MATLAB to mark the input from nodes 1 to 8. Six important nodes; nodes 2, nodes 4, nodes 5, nodes 6, nodes 7, and nodes 8, representing NANOG, SOX2, KLF4, LIN28, OCT4, and C-MYC, were selected for analysis. Nodes 1 and 3, representing KLF4 and NANOG, were excluded as they had already been considered.

## Results

### Data collection

Data on the nuclear reprogramming factors were pooled from NCBI database. The gene, its location, corresponding proteins and length were collected.

### Development of intra-networking structures, phylogenetic tree and monophyletic grouping

**Fig 1:**
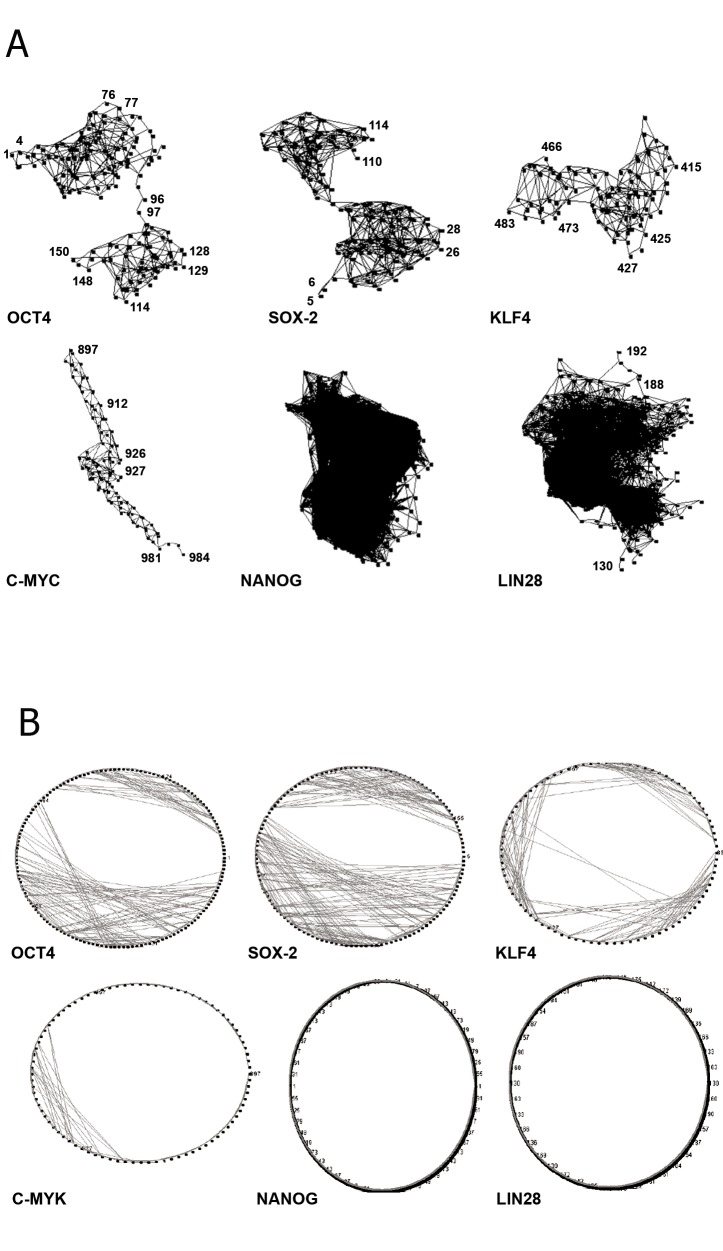
Intra-networking structures of the proteins in nuclear reprogramming factors (developed by AminoNet server). A. Show ing a 3D view of the intra-networking between amino acids for each nuclear reprogramming factor. Number represents amino acid position in network. B. 2D view of intranetworking between the amino acids of each nuclear reprogramming factor.

The intra-networking data comprised of amino acids in the transcription factors and represented in 2D and 3D view are shown in figures 1A and 1B. A 3D view of the network demonstrated that OCT4 and SOX2 comprised of two distinct halves of the network. The SOX2 had two different network clusters that were prominent. However, NANOG and LIN28 networks were dense and undifferentiated. The C-MYC formed an intra-network structure that looks like a column (Fig 1A). A 2D view of the intranetwork of C-MYC showed a minimal intranetwork. But, for NANOG and LIN28 the 2D view of the intra-network was not visible due to high density (Fig 1B).

**Fig 2:**
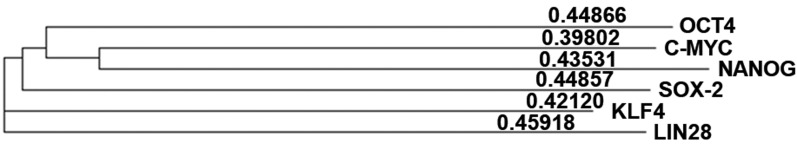
Phylogenetic tree construction of six transcription factors. This phylogenetic tree was developed using ClustalW software.

**Fig 3:**
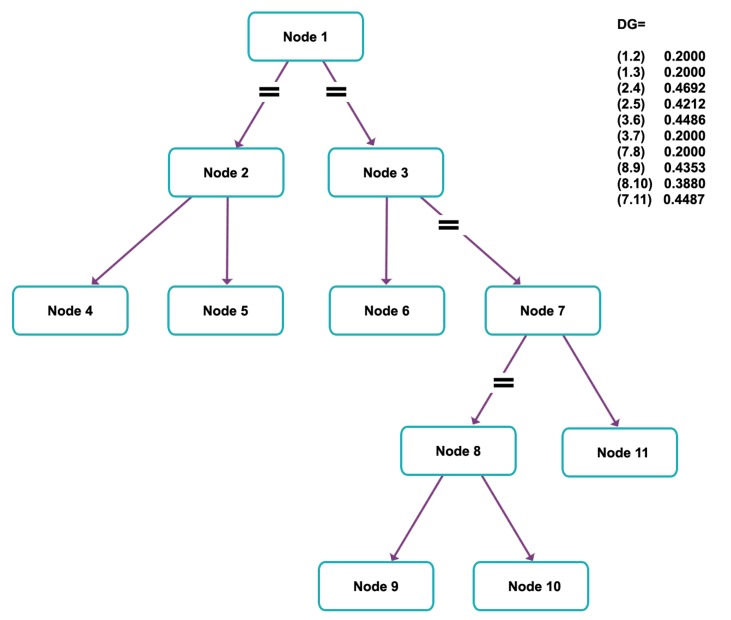
Modified phylogenetic tree with node and distance of 11 nodes (Using MATLAB). Each edge is given a weight based on the distance from the nodes. Edges which are broken (≠) imply an unknown distance between those two nodes.

The phylogram of reprogramming factors showed significant relationships among the transcription factors (Fig 2). In the tree, the length of the branches was calculated from the likelihood ratio mapping the evolutionary relationships among distinct nuclear reprogramming factors. The phylogram shows strong relationships between C-MYC and NANOG which indicated a common ancestry or the same point of evolutionary origin. Nonetheless, the sequences of OCT4, C-MYC, NANOG, and SOX2 were grouped together forming a monophyletic clade that showed a more recent common ancestor. The output of the phylogram is shown in figure 3 and the following code has been generated for the connection between nodes:

DG=sparse ([1 1 2 2 3 3 7 8 8 7], [2 3 4 5 6 7 8 9 10 11], true, 11, 11)

The above code is a sparse matrix that contains 11 nodes. The weights of each edge have been shown in figure 3.

W=[.2 .2 .46918 .42120 .44857 .2 .2 .43531 .39802 .44866];

DG=sparse ([1 1 2 2 3 3 7 8 8 7], [2 3 4 5 6 7 8 9 10 11], W)

DG=

(1,2) 0.2000
(1,3) 0.2000
(2,4) 0.4692
(2,5) 0.4212
(3,6) 0.4486
(3,7) 0.2000
(7,8) 0.2000
(8,9) 0.4353
(8,10) 0.3980
(7,11) 0.4487

To view the above inter network, the following code has been written: h = view biograph (DG); biograph object with 11 nodes and 10 edges.

After executing the above code 11 nodes interconnecting the network (Fig 3), it became a binary tree structure with each edge given a weight based on the distance from nodes. The broken edges (#) imply an unknown distance between the nodes. These were ignored (assuming the distance as .20) while network programming.

### Protein-protein network

**Fig 4:**
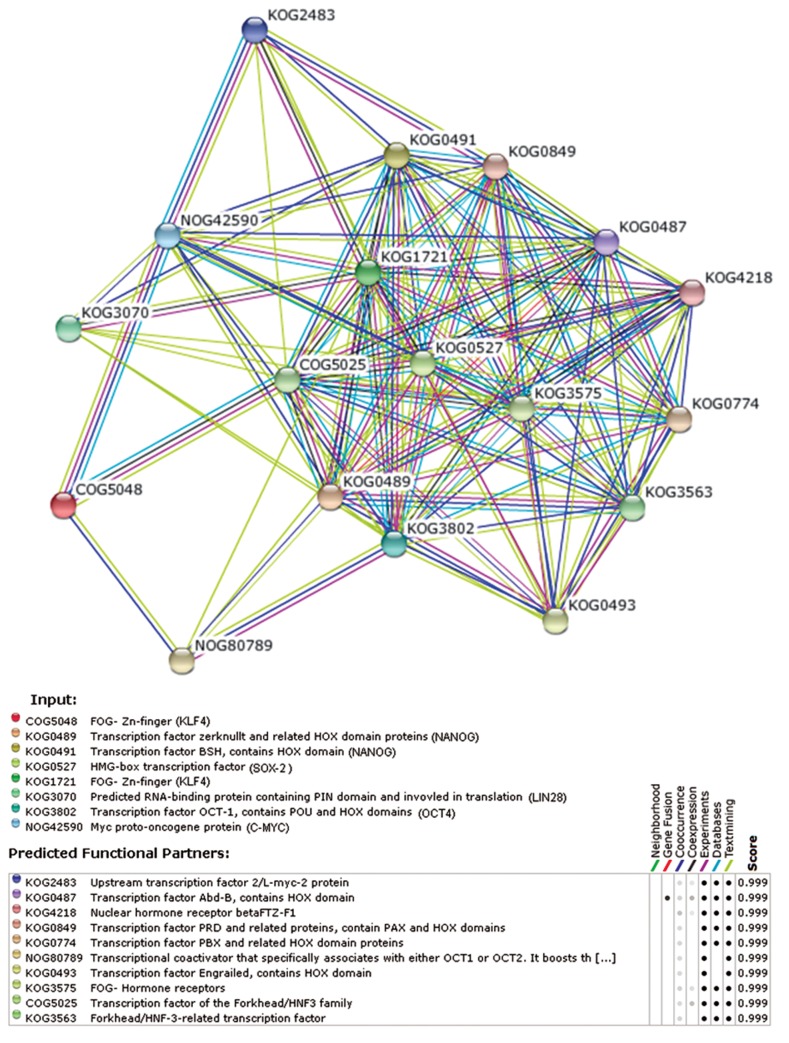
Protein-protein network design of nuclear reprogramming factors (by STRING). This network represents internetworking between six nuclear reprogramming factors.

An undirected protein-protein network between reprogramming factors, depicted in figure 4, shows that transcription factors are not only structurally interlinked, but also functionally interlink other proteins. All the nodes had a score of 0.999 therefore they are all equally important and interconnected. Furthermore, the nodes 2 plus 4 to 8 representing NANOG, SOX2, KLF4, LIN28, OCT4, and C-MYC are also composed of six common nuclear reprogramming factors.

### Development of sub-network and analysis of strongly connected components

The MATLAB programming has shown that there is a sub-network connection between nodes numbers 1 to 8. However, nodes 1 and 3, representing KLF4 and NANOG, were excluded since they were considered as nodes 5 and 2 previously. The output of the program is shown in figure S2B and the code is executed and represented as below: DG = sparse ([2 2 2 2 2 4 4 4 4 4 5 5 5 5 5 5 6 6 6 7 7 7 7 8 8 8 8], [6 7 8 5 4 7 8 3 5 2 6 7 2 8 4 3 8 2 5 3 5 4 2 5 2 6 4], true, 28,28)

**Fig 5:**
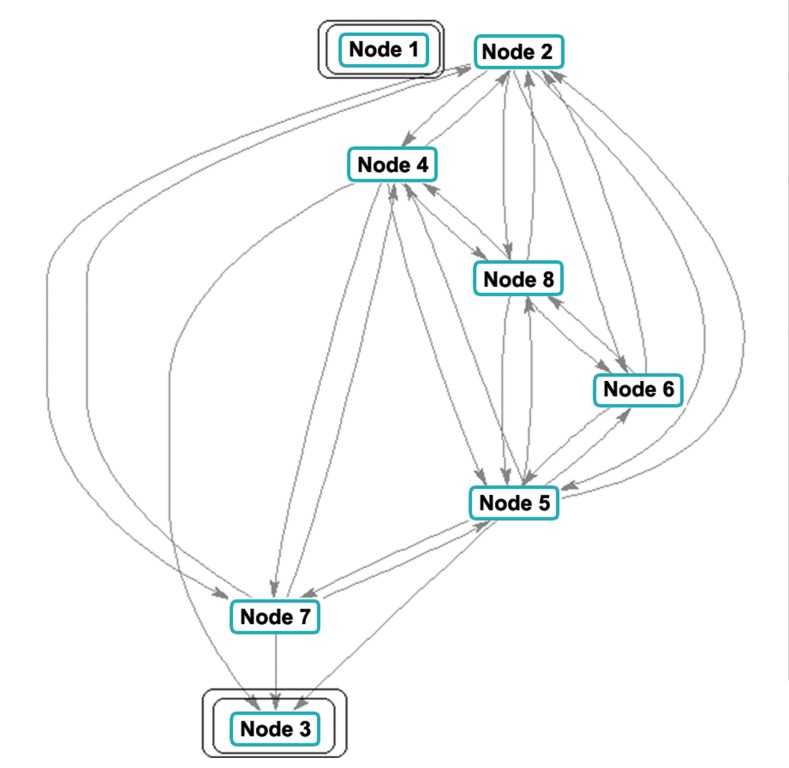
Strongly connected components in the sub-network of nuclear reprogramming factors (by MATLAB). In this figure nodes 2, nodes 4, nodes 5, nodes 6, nodes 7, and nodes 8 represent NANOG, SOX2, KLF4, LIN28, OCT4, and C-MYC. Nodes 1 and 3 were not considered since they represent KLF4 and NANOG.

DC=

(4,2) 1
(5,2) 1
(6,2) 1
(7,2) 1
(8,2) 1
(4,3) 1
(5,3) 1
(7,3) 1
(2,4) 1
(5,4) 1
(7,4) 1
(8,4) 1
(2,5) 1
(4,5) 1
(6,5) 1
(7,5) 1
(8,5) 1
(2,6) 1
(5,6) 1
(8,6) 1
(2,7) 1
(4,7) 1
(5,7) 1
(2,8) 1
(4,8) 1
(5,8) 1
(6,8) 1
>> h = view (biograph(DG));

After executing the above code, the sub-network was generated by considering the distance scores as 1 as STRING scores showing .9999 (Fig 5). As shown by MATLAB, the node colors indicated strongly connected components between the nuclear reprogramming factors, which indicated strong relations among the connected components as per the color. The source code is: >> [S, C] = graphconncomp(DG)

S =

23

C =

Columns 1 through 26

1 3 2 3 3 3 3 3 4 5 6 7 8 9 10 11 12 13 14 15 16 17 18 19 20 21

Columns 27 through 28

22 23

>> colors = jet(S);

for i = 1:numel(h.nodes)

h.Nodes(i).Color = colors(C(i),:);

end

>>

The algorithm for the strongly connected component was generated following Cormen et al. (29) for the nuclear reprogramming factors, which is as follows:

STRONGLY-CONNECTED-COMPONENT (G)

1. Calls DFS(G) to calculate the finishing time f[u] for each vertex
2. Next to compute the transpose of the GT
3. Call DFS(GT ), but in the main loop of DFS, considering the vertices in order of decreasing f[u] ( as computer in line 1)
4. Output the vertices of each tree in the depth-first forest formed in the line 3 as a separate strongly connected component.

G^T^ stands for transpose of graph G (DEPTH FIRST SEARCH as DFS). The output of the program shows the same color of each of the nodes (nodes 2, 4, 5, 6, 7, 8), This indicates that each of the six nodes are equally important.

## Discussion

At present, protein network analysis demands the use of computational biology to enhance predictions of protein–protein interactions (30) and visualization (31). However, as shown in the above intra-networking, the 3D view of NANOG and LIN28 show a dense and undifferentiated network that forms a cluster as a result of the location of the amino acid. With the help of MATLAB, a directed network using a simple directed graph was created (32) where NANOG and C-MYC were situated at the leaf node. Using STRING, an undirected protein-protein network was generated that showed all proteins strongly connected by physical and functional interactions. Therefore, from the bioinformatics stand point, it can be stated that these six proteins should be put in one group with the title 'nuclear reprogramming group of proteins for iPS cell generation'.

The first experimental evidence regarding nuclear reprogramming, reported by Briggs and King (33), came from the reprogramming of Rana pipiens to generate normal tadpoles. In last few decades, three significant advances in "Cellular Reprogramming" have been developed that include the isolation of stem cells from embryos, animal cloning by nuclear transfer, and induced pluripotent stem cells (34). However, the nuclear reprogramming of somatic cells is a new idea, as demonstrated by Takahashi et al. in 2007, when they showed that mouse and human fibroblasts could be reprogrammed through the nuclear reprogramming to generate iPS cells with similar qualities to embryonic stem (ES) cells (1,5). This discovery has opened a new basis on which to use pluripotent cells for drug discovery, cell therapy and basic research. Scientists consider iPS cells as a major development in stem cell research as they give new insights into the pathways involved in the maintenance of pluripotency (35).

The four reprogramming factors (OCT4, SOX2, NANOG, and LIN-28) also known as 'Yamanaka Factors' (36) have been widely used to reprogram somatic cells into iPS cells (37). In fact, the six common nuclear reprogramming factors (OCT4, SOX2, C-MYC, KLF4, NANOG, and LIN28) have become a point of attention in the present revolution of iPS cells. Nonetheless, the reprogramming mechanism has not been unidentified to date so it has become an important research topic. However, some questions still remain to be answered: Are the six transcription factors evolutionarily linked? Are there any inter-network connections between the transcription factors? Which reprogramming factor is important for the generation of iPS cells? How are the amino acids interlinked with each other in a particular protein?

Our analyses using intra- and inter-network development has clarified these impending queries with a hypothetical answer. On the other hand, Jaenisch and Young (38) have proposed a regulatory cartoon that shows a hypothetical regulatory network between the transcription factors for signal transduction pathways. We have proposed an in silico relationship between the six nuclear reprogramming factors (39) and at this juncture, we have developed intra-and inter-networks, which is significant. According to Viswanathan and Daley (40), all the currently described reprogramming factors-OCT4, SOX2, KLF4, C-MYC, NANOG, and LIN28- have been associated with oncogenesis. Probably, this phenomenon is not a coincidence and there may be relations between them. Expression of the reprogramming factors in the ischemic cell commences a sequence of stochastic events that may result in nuclear reprogramming leading to iPS cells, a pathway supported by Mikkelsen et al. (41). They state that the activation of transcription factors for pluripotency can occur at different times after infection in the fibroblast. Therefore the expression of transcription factors may cause the initiation of a sequence of epigenetic events, like chromatin modifications or changes in DNA methylation, generating pluripotent phenomena (33).

## Conclusion

This paper has shown an innovative and rapid method for the analysis of intra and internetworks between the nuclear reprogramming factors. *In vitro* nuclear reprogramming for the generation of iPS cells is a complex phenomenon where the transcription factors play a crucial regulatory network. To date, the existence of a regulatory network between the proteins for the reprogramming of somatic cells to iPS cells remains unknown. Therefore this protein group, the transcription factors for iPS cell generation, can be deemed a new group of proteins titled 'nuclear reprogramming group of proteins for iPS cell generation'. The data presented in this paper may be helpful to researchers trying to understand the complex regulatory network governing iPS cell generation.
